# Association between the food security status and dietary patterns with polycystic ovary syndrome (PCOS) in overweight and obese Iranian women: a case-control study

**DOI:** 10.1186/s13048-021-00890-1

**Published:** 2021-10-13

**Authors:** Mahtab Badri-Fariman, Amirmansour Alavi Naeini, Khadijeh Mirzaei, Ashraf Moeini, Mostafa Hosseini, Seyedeh Elaheh Bagheri, Milad Daneshi-Maskooni

**Affiliations:** 1 Department of Community Nutrition, School of Nutritional Sciences and Dietetics, Tehran University of Medical Sciences, Tehran, Iran; 2 Department of Community Nutrition, School of Nutrition and Food Sciences, Isfahan University of Medical Sciences, Isfahan, Iran; 3 Department of Obstetrics and Gynecology, School of Medicine, Tehran University of Medical Sciences, Tehran, Iran; 4 Department of Biostatistics and Epidemiology, School of Public Health, Tehran University of Medical Sciences, Tehran, Iran; 5 School of Paramedicine, Guilan University of Medical Sciences, Langroud, Iran; 6 Department of Nutrition, School of Medicine, Jiroft University of Medical Sciences, Jiroft, Kerman Iran

**Keywords:** Food insecurity, Dietary patterns, Polycystic ovary syndrome, Obesity, Overweight

## Abstract

**Background:**

Polycystic ovary syndrome (PCOS), as one of the significant endocrine disorders, is common among women worldwide. Food insecurity (FI) and unhealthy dietary patterns can negatively affect reproductive health. The effects of the lifestyle modifications, especially dietary components, on PCOS are contradictory. The aim was the assessment of association between PCOS with food security status and dietary patterns among overweight or obese women.

**Methods:**

This case-control study was performed on 240 overweight and obese women with and without PCOS (ratio 1:1) referred to the infertility clinic of Arash Hospital, Tehran, Iran. The general and socioeconomic characteristics, anthropometrics (weight, height, body mass index (BMI), waist circumference, hip circumference), physical activity, food security status, and dietary intakes (or patterns) were assessed using valid questionnaires, scales, stadiometer, and tape meter. The significant *p*-value was < 0.05.

**Results:**

The prevalence of FI was 60% in women with PCOS and 30% in healthy women. PCOS risk was positively related to FI, quasi-western dietary patterns, low economic levels, waist circumference, and menstrual age and negatively with physical activity and healthy dietary patterns, even after controlling the potential confounders (*P* <  0.05). PCOS women had a higher intake of saturated fats, monounsaturated fats, oleic acid, fluorine, sucrose, and caffeine and a lower intake of vitamins A, B_5_, B_6_, B_12_, C, and D, potassium, proteins, carbohydrates, cholesterols, docosahexaenoic acid, potassium, carotenes, lutein, beta-cryptoxanthin, lycopene, calcium, iron, thiamine, riboflavin, niacin, tetra- and dihydrofolate, biotin, phosphorus, magnesium, zinc, copper, fiber (total, insoluble, and crude), glucose, galactose, fructose, and lactose compared to the healthy women (*P* <  0.05).

**Conclusions:**

FI, quasi-western dietary patterns, low economic levels, and waist circumference were significantly associated with the higher risk of PCOS. The lifestyle changes, especially dietary patterns, may be an essential strategy for reducing PCOS. Further studies are warranted to confirm these findings and to identify the underlying mechanisms.

## Introduction

Polycystic ovary syndrome (PCOS), as one of the main health challenges worldwide [1], is the most prevalent and complex type of endocrine disorder among women of reproductive age [2–7]. According to Rotterdam criteria, this syndrome affects 19.5% of Iranian women [8, 9]. PCOS is characterized by polycystic ovarian morphology (PCOM) [3, 10], ovulatory dysfunction [4, 10–12], menstrual disorders [2, 4, 13], reproductive problems, infertility [3, 4, 14, 15], oligomenorrhea [12, 15], hyperandrogenism [2, 4, 10–12, 15], and some clinical manifestations of alopecia, acne, oily skin, and hirsutism [2, 3, 12–14]. Moreover, several complications including insulin resistance (IR) [9, 12–14, 16–18], type 2 diabetes (T2DM) [11–13, 18, 19], cardiovascular disease (CVD) [3, 9, 11, 13, 14, 17], endometrial cancer [3, 11, 19], mental and behavioral disorders (e.g., anxiety, depression, and lack of self-confidence) [12, 20], dyslipidemia [13, 14, 16, 18], metabolic syndrome [9, 17, 18], and specifically obesity [13, 14, 16] are associated with not-treated PCOS. It is estimated that nearly 40-60% of PCOS women are overweight or obese [2].

Generally, the etiology of PCOS is very complex and not well clear, but refers to multifactorial causes, which can be genetic or modifiable factors, such as environment [2, 8, 9, 21, 22] and lifestyle factors, such as smoking [2], physical activity [2, 3, 23], and diet [2, 8, 9, 21, 23]. However, inappropriate lifestyle, particularly unhealthy dietary patterns, result in IR and obesity [8, 24], considered as most common etiological factors of this syndrome [3, 25]. So, lifestyle modifications, especially considering reducing IR and obesity, may also have a vital role in treating PCOS [1].

Dietary patterns have potential effects on IR and overweight [26, 27] and can influence the expression of genes involved in critical metabolic pathways [28]. It is indicated that Iranian women with a higher risk of PCOS had a higher intake of western dietary patterns and less plant-based diets [8]. Qualitative or quantitative dietary deficiencies have been reported to be related to the higher weight that can lead to the occurrence or progression of PCOS [29].

In addition, food insecurity (FI) is considered as a condition that there is limited or uncertain access to adequate healthy diets, which can cause a broad spectrum of socio-emotional issues, obesity, and chronic disorders [30, 31] that may be affected by several socio-economic factors [32]. It was found that FI itself can contribute to the disturbance of the eating patterns and thereby the dietary intakes [33]. Nowadays, almost 795 million persons suffer from FI around the world [34]. It is estimated that 25 and 50% of Iranian women have energy restriction and nutritional deficiencies, respectively [35]. Since the prevalence of FI in Iran is increasing, preventive approaches are needed to reduce their subsequent adverse results [36].

Evidence suggests that a combination of the nutritional components in the form of healthy dietary patterns have significant beneficial effects on both prevention and treatment of PCOS [37] since their potential impact on the numbers of metabolic and inflammatory factors [38]. Dietary approaches to stop hypertension (DASH) diet as a kind of dietary pattern which is rich in whole grains, vegetables, fruits, and low-fat dairy products and low in carbohydrates, saturated fats, and cholesterol, has beneficial effects on the BMI [18, 39], antioxidant status [39], IR, and nitric oxide [18] in overweight or obese PCOS women. A recent study demonstrated the negative association between the severity of inflammatory profiles, IR, and hyperandrogenemia with the Mediterranean diet (MD) in women with PCOS [40]. Negative and positive associations were reported between the intakes of high glycemic index (GI) diets and anti-inflammatory dietary patterns with PCOS risk, respectively [9]. Also, it was reported that total dietary intake of protein and energy intake of simple sugars are significantly lower in PCOS than in healthy women [41]. However, there were no significant differences in the dietary intakes of PCOS and healthy women in another study [42]. Babapour et al. negatively indicated the relationship between serum levels of magnesium with the development of PCOS among overweight or obese women [10]. Although, another researcher failed to find any positive effects of magnesium supplementation on the serum lipids and glycemic indicators [43]. On the other hand, supplementation with dietary intakes of fiber and magnesium was related to lower IR and hyperandrogenemia [44]. However, the optimal diet is not yet well-understood and its overall impact on the risk of PCOS mainly remains unknown [1, 19].

Given that, no study has assessed the relationship between both food security status and dietary patterns with PCOS. Therefore, we aimed to investigate the association between PCOS with food security status and dietary patterns of overweight and obese Iranian women referred to the infertility clinic in Tehran, Iran.

## Methods

### Study design and population

This case-control study was carried out on overweight and obese women referred to the infertility clinic of Arash Hospital, Tehran, Iran. The inclusion criteria of the cases included females with the diagnosis of PCOS based on the presence of PCOM on ultrasound according to the doctor’s confirmation or based on the standard Rotterdam diagnosis [45] during less than 6 months and without any receiving treatment before the study, age between 20 and 48 years, and BMI equal to or more than 25 kg/m^2^. The inclusion criteria of the controls included females without PCOS diagnosis, with normal 26-33 days’ menstrual pattern, age between 20 and 48 years, and BMI equal to or more than 25 kg/m^2^. The exclusion criteria for both groups included the current history of cardiovascular, liver, and kidney diseases, smoking, taking drugs that can affect the metabolism of hormones and body composition, having strenuous physical activities, and not consent to participate in the study.

At first, a pre-test was performed on women with PCOS (*n* = 20) and healthy non-PCOS women (*n* = 20) to get them acquainted with the research environment, manner of responding to questionnaires, estimating sample size, and accuracy of the study. According to the performed pre-test, the percentage of FI was obtained 30% (P_1_ = 0.30) for non-PCOS women and 55% (P_2_ = 0.55) for PCOS women. The required sample size was calculated 102 according to the following formula.

$$\mathrm{n}=\frac{2{\left({\mathrm{Z}}_{1\hbox{-} \upalpha /2}+{\mathrm{Z}}_{1\hbox{-} \upbeta}\right)}^2\times \left[\overline{\mathrm{P}}\left(1\hbox{-} \overline{\mathrm{P}}\right)\right]}{{\left({\mathrm{P}}_1+{\mathrm{P}}_2\right)}^2}$$

$${\mathrm{P}}_1=30\%,{\mathrm{P}}_2=55\%,\upalpha =\upbeta =0.05$$

$${\mathrm{P}}_1=30\%,{\mathrm{P}}_2=55\%,\upalpha =\upbeta =0.05$$

**Fig. 1:**
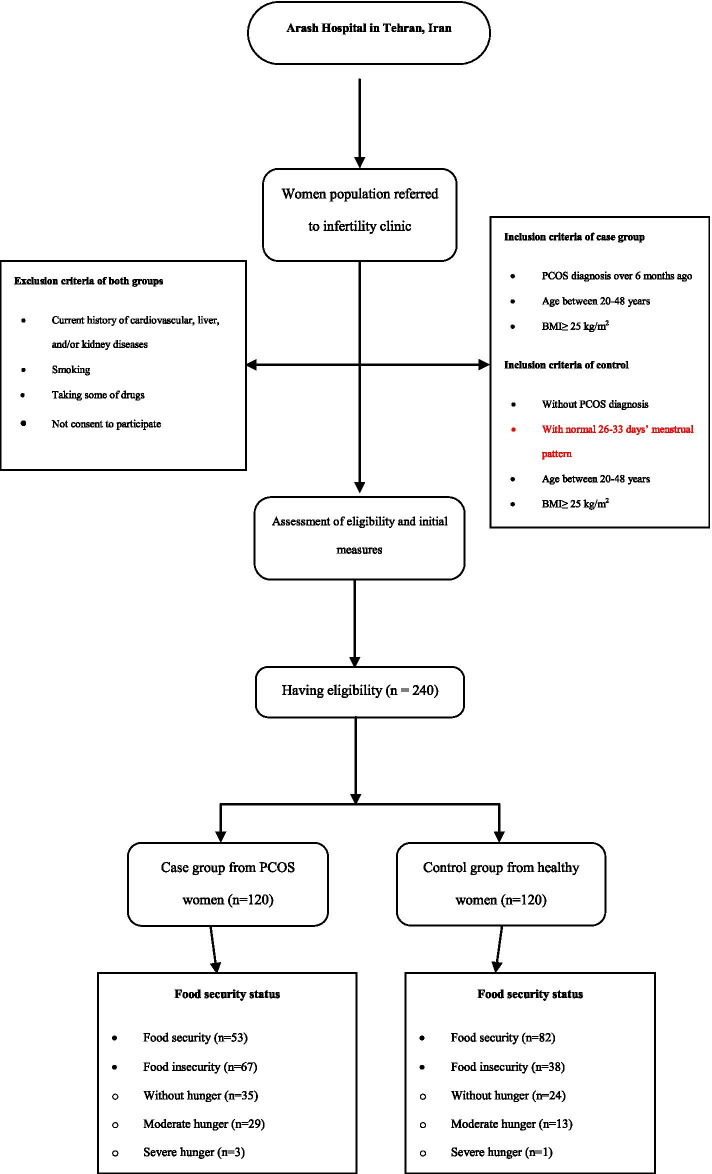
Study flowchart

Finally, considering the reliability and probability of sample loss, the sample size was determined 120. Since the dietary patterns are affected by FI, it was considered the main factor for calculating the sample size. Data analysis was conducted on 240 women who met the criteria, including 120 women with a definitively PCOS diagnosis as the case group and 120 similar healthy women as the control group (Fig. 1).

### Data collection

After full explanations of the goals and methods of the study and receiving the written consent form, data on general characteristics (anthropometry and socio-economic factors), physical activity, food security status, and dietary patterns were collected from all participants using valid and reliable questionnaires through face-to-face interviews and some measures.

### General characteristics

Data on anthropometry and socio-economic factors were collected using the general questionnaire included questions about age, ethnicity, education and economic levels, job status, number of family members and employed persons of household, insurance support, house ownership, and marital status, menstrual age, and number of pregnancies and children. Also, additional assessment in the case group was performed using a self-reported questionnaire that included some signs and symptoms of PCOS. The participants’ weight was measured using a Seca scale with minimal clothing and without any shoes with an accuracy of 0.1 kg (kg) and height was measured using a wall-attached tape meter with an accuracy of 0.5 cm (cm). Then BMI was calculated by dividing weight in kilograms by the square of height in meters (kg/m^2^) for each woman. Also, the waist circumference was measured between the chest and hips with light clothing with an accuracy of 10 cm without any pressure on the pelvic range. The hip circumference was calculated using the same route with an elastic meter in the broadest part of the hips [46].

### Physical activity

Data on the physical activity levels was collected using the metabolic equivalents (MET)-based questionnaire, which its validity and reliability have been approved in Iran [47]. The results were presented as MET with nine levels of activity from lowest activity, such as rest and sleep (MET equal to 0.9) to intense activity, such as jogging and basketball (MET more than 6). Women were classified into three categories of physical activity, including low (MET less than 3), moderate (MET between 3 and 6), and severe (MET more than 6) [48]. Then the daily energy expenditure of each woman was calculated based on their weight and MET-hour per day.

### Food security status

The 18-item United States Department of Agriculture (USDA) food security questionnaire which has already been validated in Iran [49], was used to determine the FI status of households over the past 12 months. As presented in Table 1, the participants were stratified into two groups based on their scores from positive answers (score 1) or negative answers (score 0). In this regard, positive answers were considered as “often, sometimes, almost every month, some months, and yes” and negatives answers as “not correct, refused or did not know, only once or twice a month, and no”. Also, the previous-related questions, in which the participants have received a score of 0, were not asked and were given a score of 0. The maximum score of this questionnaire would be 14 if both sections were completed [50].

**Table 1:** The status of household food security according to USDA FI questionnaire

			Code	With children < 18-y^**a**^	Without children < 18-y^**b**^
Household food security status	Secure		0	0-2	0-2
	Insecure	Without hunger	1	3-7	3-5
		Moderate hunger	2	8-12	6-8
		Severe hunger	3	13-18	9-10

^a^Number of positive answers out of 18 scores

^b^Number of positive answers out of 10 scores

### Dietary intakes

Data on the dietary intakes of women was collected using a semi-quantitative food frequency questionnaire (FFQ) that has been previously validated in Iran [51]. The FFQ consisted of 168 food groups with standard size units of foods and beverages commonly consumed in the dish of Iranian foods. According to their food compositions, items were categorized into 19 groups to analyze of the dietary patterns [52]. Based on the types of foods, participants reported the frequency of food intake as never, per day, week, month, and year over the past year before this study [53]. Significantly, the case group was asked for their intake before the definite diagnosis of PCOS. Then all data on dietary assessments were converted to grams via Iranian household measures.

### Statistical analysis

Quantitative variables using the t-test method were presented as the mean (± standard deviation; SD), and qualitative variables using the chi-square method were presented as the number (%) between two groups. All of the significant variables were entered into the multivariate logistic regression model, and final independent variables were identified using the backward method after adjusting for confounders. The factor analysis model of main dietary components with the Varimax rotation was used for each classified food group to determine dietary patterns. Then main dietary patterns were entered into the univariate logistic regression model separately. Finally, PCOS-related dietary patterns and other significant variables (except food security) in the univariate analysis models separately were entered into the multivariate logistic regression model with the forward method for determining the final independent risk factors and controlling the potential confounders. Statistical analysis was performed using the SPSS software (version 16), Nutritionist IV (First Databank, Hearst Corp., San Bruno, CA, USA), and Stata11SE software. *P* values less than 0.05 were considered statistically significant.

## Results

The frequency of PCOS symptoms among case group including menstrual disorders was 90% (*n* = 108), acne was 52.5% (*n* = 63), oily skin was 48.3% (*n* = 58), hirsutism was 47.5% (*n* = 57), and other symptoms were 0.8% (*n* = 1).

Table 2 shows the food security status, anthropometry, and socio-economic factors of both case (*n* = 120) and control (*n* = 120) groups. The prevalence of food security was significantly lower in the case group than in the controls (*P* <  0.001). Approximately 60% of PCOS women (*n* = 67) had FI, and nearly half of them (*n* = 32) experienced FI with hunger. While only 30% of the control group (*n* = 38) had FI, and the majority of them (*n* = 24) had no hunger (Fig. 1). According to this table, the case group had significantly higher menstrual age (13.48 ± 1.97 vs. 12.82 ± 1.43) and waist circumference (100.65 ± 12.04 vs. 97.61 ± 5.37) and lower economic levels, rest or sleep MET-hour score (1.32 ± 0.99 vs. 1.37 ± 0.13), and the numbers of pregnancies, children, and family members compared to the controls (*P* <  0.05). There were no significant differences between ethnicity, weight, height, BMI, education level, job status, insurance support, house ownership, marital status, numbers of employed persons of household, and hip circumference in PCOS and healthy women (*P* > 0.05).

**Table 2:** Characteristics of the participants according to the T-test and chi-square test

			Cases (***n*** = 120)	Controls (***n*** = 120)	***P*** value
Food security	Secure		53 (44.2%)	82 (68.3%)	< 0.001
	Insecure	Without hunger	35 (29.2%)	24 (20%)	
		Moderate hunger	29 (24.2%)	13 (10.8%)	
		Severe hunger	3 (2.5%)	1 (0.8%)	
Ethnicity	Fars		58 (48.3%)	47 (39.2%)	0.12
	Turk		35 (29.2%)	50 (41.7%)	
	Others		27 (27.5%)	23 (19.2%)	
Weight (kg)			76.83 (±10.89)	74.75 (±8.12)	0.096
Height (cm)			161.16 (±5.56)	160.70 (±7.03)	0.57
BMI (kg/m^2^)			29.55 (±3.70)	28.88 (±1.74)	0.076
Education level	Under diploma		67 (55.8%)	65 (54.2%)	0.79
	Diploma and higher		53 (44.2%)	55 (45.8%)	
Economic level	Upper middle to high		78 (65%)	99 (82.5%)	0.002
	Lower middle to poor		42 (35%)	21 (17.5%)	
Job status	Unemployed		80 (66.7%)	80 (66.7%)	0.79
	Free, worker, or other		20 (16.7%)	23 (19.2%)	
	Employee		20 (16.7%)	17 (14.2%)	
Number of employed persons of household	1		86 (71.7%)	86 (77.7%)	0.78
	≥ 2		34 (28.3%)	34 (28.3%)	
Marital status	Unmarried or others		17 (14.2%)	19 (15.8%)	0.71
	Married		103 (85.8%)	101 (84.2%)	
Number of pregnancies	0		71 (59.2%)	52 (43.3%)	0.002
	≥ 1		49 (40.8%)	68 (56.7%)	
Number of children	0		88 (73.3)	59 (49.2%)	< 0.001
	≥ 1		32 (26.7%)	61 (50.8%)	
Menstrual age (year)			13.48 (±1.97)	12.82 (±1.43)	0.003
Waist circumference			100.65 (±12.04)	97.61 (±5.37)	0.013
Hip circumference			117.22 (±12.04)	118.25 (±5.15)	0.39
Score of rest or sleep (MET-hour)			1.32 (±0.99)	1.37 (±0.13)	< 0.001
Number of family members	≤ 2		68 (56.7%)	39 (32.5%)	< 0.001
	≥ 3		52 (43.3%)	81 (67.5%)	
House ownership	Personal or free		41 (34.2%)	55 (45.8%)	0.065
	Rental or pawn		79 (65.8%)	65 (54.2%)	
Insurance support status	Medical services (Health Insurance)		18 (15%)	33 (27.5%)	0.055
	Social security organization		77 (64.2%)	68 (56.7%)	
	other		25 (20.8%)	19 (15.8%)	

Data are mean (±SD) for quantitative variables and number (%) for categorical variables

*MET* Metabolic equivalents, *BMI* Body mass index, *kg* kilogram, *cm* centimeter

**Fig. 2:**
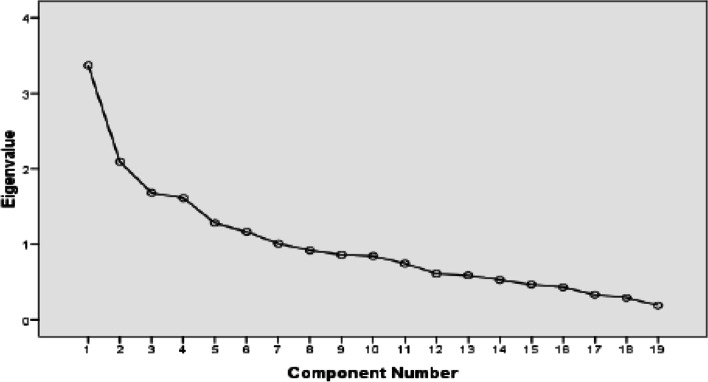
Scree Plot diagram of identifying main dietary patterns

Also in this study, two main healthy and quasi-western dietary patterns were defined (Fig. 2) and accounted for 24% of the whole variances that healthy dietary patterns had a higher rate than the quasi-western ones. As shown in Table 3, the quasi-western dietary patterns including sugars, sweets, desserts, industrial juice and soft drinks, processed meats, red and organ meats, refined grains, salt, French fries and potato chips, and tea and coffee had the highest factor loading, respectively. Solid oils, animal fat, and salt had negative factor loadings. The PCOS group had a significantly higher intake of sugars, sweets, and desserts (28.47 ± 4.50 vs. 9.49 ± 4.12), industrial juice and soft drinks (8.77 ± 2.77 vs. 4.77 ± 3.12), processed meats (15.85 ± 4.26 vs. 3.70 ± 1.64), red and organ meats (17.62 ± 2.84 vs. 10.40 ± 2.47), salt (4.90 ± 1.77 vs. 3.32 ± 1.24), and tea and coffee (21.24 ± 3.20 vs. 8.17 ± 4.62) compared to the control group (*P* <  0.05).

**Table 3:** Dietary patterns based on the T-test and factor loading matrix of two main dietary patterns with the Varimax rotation

Food groups	Major food items in Iran	Dietary patterns^**a**^		Cases (***n*** = 120)	Controls (***n*** = 120)	***P*** value
		Pattern 1: healthy	Pattern 2: quasi-western			
Processed meats	Sausage, kielbasa, hamburger	–	0.465	15.85 (±4.26)	3.70 (±1.64)	< 0.001
Red and organ meats	Beef and veal, mutton, minced meat, heart, liver, and offal, by-products and abomasum, kale pache, tongue, brain	–	0.456	17.62 (±2.84)	10.40 (±2.47)	< 0.001
Fish and poultry	Types of fish, tuna, chicken	0.556	–	24.81 (±5.83)	37.59 (±5.58)	< 0.001
Egg	Egg	0.465	–	13.78 (±5.25)	22.66 (±5.61)	< 0.001
Dairy products	Types of milk (low, normal, or high fat), yogurt (low, normal, or high fat), creamy or dripped yogurt, dough, chocolate milk, cheese (creamy or normal), types of ice cream, curd, cream, butterfat	0.595	–	2.52 (±1.58)	5.69 (±1.17)	< 0.001
Tea and coffee	Tea and coffee	–	0.319	21.24 (±3.20)	8.17 (±4.62)	< 0.001
Fruits and vegetables	Cantaloupe and watermelon, melon, watermelon, pear, apricot, cherry and sour cherry, apple, peach, nectarine, green tomato, grape, kiwi, grapefruit, orange, persimmon, tangerine, pomegranate, yellow or red plum, strawberry, banana, lemonade, lemongrass, raisins, fresh berries, fresh figs, compotes, dried figs, dates, dried berries, peach and apricot leaves, natural juices, other fruits	0.797	–	232.91 (±13.33)	406.37 (±14.28)	< 0.001
	Lettuce, cucumber, stewed vegetables, eggplant, celery, green peas, green beans, green peppers, bell peppers, turnips, squash, pumpkin stew, mushrooms, raw or fried onions, boiled potatoes, garlic, Spinach, raw or cooked carrots, other vegetables					
Industrial juice and soft drinks	Industrial juice, lemon juice, types of drinks (soft drinks, water poetry, industrial syrups)	–	0.714	8.77 (±2.77)	4.77 (±3.12)	< 0.001
Legumes and soy	Beans, lentils, chickpeas, cotyledon, broad bean, mung bean and other legumes, soybean meal	0.429	–	171.72 (±12.53)	248.38 (±12.06)	< 0.001
Nuts	Peanuts, almonds, walnuts, pistachios, hazelnuts, any kind of seed	0.351	–	65.34 (±4.19)	79.05 (±6.41)	0.042
Whole grains	Sangak bread, taftoon, barbari, barley bread, barley, wheat, oatmeal, corn	–	–	1228.03 (±57.18)	1333.29 (±55.88)	0.149
Refined grains	Bread (milk, fried, oily, or sugar bread), lavash, baguette, wheat flour, rice, pasta, noodles, vermicelli, crackers	–	0.392	271.78 (±17.76)	247.14 (±16.80)	0.120
French fries and potato chips	French fries, potato chips	–	0.315	2.52 (±0.58)	0.38 (±0.04)	0.184
Sugars, sweets and desserts	Types of cakes, dry or wet sweets, chocolates, homemade halva, puff pastry, biscuits, donuts, caramel cream, sugar, sugar or cheese, candy, honey, jams, sugary halva, quotes, candy, turmeric, sohan	–	0.788	28.47 (±4.50)	9.49 (±4.12)	< 0.001
Pickles and salinity	Pickles and salinity	–	–	219.41 (±17.96)	222.96 (±13.38)	0.894
Solid oils and animal fat	Margarine, butter, solid vegetable oil, animal oil, tallow, mayonnaise	−0.454	–	102.66 (±9.17)	56.30 (±8.36)	< 0.001
Liquid oils	Types of liquid, olive, or green oil	0.479	–	25.99 (±7.19)	32.01 (±5.49)	0.059
Tomato	Tomatoes, red sauce	0.701	–	36.33 (±7.99)	471.29 (±5.37)	< 0.001
Salt	Table salt, food salt	−0.392	0.376	4.90 (±1.77)	3.32 (±1.24)	< 0.001

All data are mean (±SD) for quantitative variables

^a^Factor loadings< 0.30 were not listed in the table for simplicity

On the other hand, healthy dietary patterns including fruits and vegetables, tomato, dairy products, fish and poultry, liquid oils, egg, legumes and soy, and nuts had the highest factor loading, respectively. According to Table 3, the intake of fruits and vegetables (232.91 ± 13.33 vs. 406.37 ± 14.28), tomato (36.33 ± 7.99 vs. 471.29 ± 5.37), dairy products (2.52 ± 1.58 vs. 5.69 ± 1.17), fish and poultry (24.81 ± 5.83 vs. 37.59 ± 5.58), egg (13.78 ± 5.25 vs. 22.66 ± 5.61), legumes and soy (171.72 ± 12.53 vs. 248.38 ± 12.06), and nuts (65.34 ± 4.19 vs. 79.05 ± 6.41) were significantly lower in the PCOS group compared to the controls (*P* <  0.05).

Regarding dietary intakes of micro and macronutrients, PCOS women had significantly higher food consumption and higher intake of saturated fats, monounsaturated fatty acids (MUFAs), oleic acid, fluorine, sucrose, and caffeine compared to the controls (*P* <  0.05). While non-PCOS women had a significantly higher intake of vitamins A, B_5_, B_6_, B_12_, C, and D, potassium, proteins, carbohydrates, cholesterols, docosahexaenoic acid, potassium, beta- and alpha-carotene, lutein, beta-cryptoxanthin, lycopene, calcium, iron, thiamine, riboflavin, niacin, tetra- and dihydrofolate, biotin, phosphorus, magnesium, zinc, copper, fiber (total, insoluble, and crude), glucose, galactose, fructose, and lactose compared to the cases (*P* <  0.05). There were no significant differences between the intake of calories, trans fats, polyunsaturated fatty acids (PUFAs), linoleic acid, linolenic acid, eicosapentaenoic acid, sodium, vitamin E, alpha-tocopherol, manganese, selenium, chromium, soluble fiber, total sugar, and maltose between the groups (*P* > 0.05).

Tables 4 and 5 show odds ratios (ORs) and confidence intervals (CIs) of the association between important independent risk factors with PCOS. According to the final analysis model, after controlling the potential confounders, there were significant positive associations between the risk of PCOS with FI (OR = 2.665, 95% CI = 1.461-4.860), waist circumference more than 97 cm (OR = 2.262, 95% CI = 1.301-3.933), and MET score equal to or less than 1.33 per hour (OR = 2.165, 95% CI = 1.244-3.769) (*P* <  0.05, Table 4). According to the multivariate logistic regression model, PCOS risk was positively associated with the quasi-western dietary patterns (OR = 51.890, 95% CI = 18.140-148.43), low economic levels (OR = 6.886, 95% CI = 2.745-17.275), menstrual age (OR = 1.409, 95% CI = 1.128-1.760), and waist circumference (OR = 1.041, 95% CI = 1.001-1.082) and negatively associated with healthy dietary patterns (OR = 0.140, 95% CI = 0.085-0.230) and MET score (OR = 0.008, 95% CI = 0.000-0.194) (*P* <  0.05, Table 5).

**Table 4:** Final analysis model of the association between food security and other important risk factors with PCOS

Factors		OR (95% CI)	***P*** value
Food security	Insecure	1	0.001
	Secure	2.665 (1.461-4.860)	
Waist circumference (cm)	≤ 97	1	0.004
	> 97	2.262 (1.301-3.933)	
MET-hour	> 1.33	1	0.006
	≤ 1.33	2.165 (1.244-3.769)	

*Abbreviations*: *OR* Odds ratio, *CI* Confidence interval, *cm* centimeter, *MET* Metabolic equivalents

**Table 5:** Association between dietary patterns and other important risk factors with PCOS

Factors		OR (95% CI)	β^**a**^	***P*** value
Dietary patterns	Healthy	0.163 (0.103-0.256)^b^	−1.817^b^	< 0.001
		0.140 (0.085-0.230)^c^	−1.969^c^	
	Quasi-western	32.754 (13.249-80.973)^b^	3.489^b^	< 0.001
		51.890 (18.140-148.43)^c^	3.949^c^	
Waist circumference (cm)		1.041 (1.001-1.082)	0.040	0.043
Menstrual age (year)		1.409 (1.128-1.760)	0.343	0.003
Low economic level		6.886 (2.745-17.275)	1.930	< 0.001
MET-hour		0.008 (0.000-0.194)	−4.791	0.003

*Abbreviations*: *OR* Odds ratio, *CI* Confidence interval, *cm* centimeter, *MET* Metabolic equivalents

^a^β is a standardized regression coefficient that its negative value indicates a risk reduction

^b^Association between the dietary patterns and PCOS according to the univariate analysis models and ^c^multivariate logistic regression model

## Discussion

To our best knowledge, this is the first case-control study determining the association between food security status and dietary patterns with PCOS. The results demonstrated that FI, especially with moderate to severe hunger, was significantly higher among PCOS women than healthy women. Moreover, PCOS risk was positively associated with FI, quasi-western dietary pattern, low economic levels, waist circumference, and menstrual age and negatively associated with physical activity and healthy dietary patterns even after controlling the potential confounders.

According to our result, almost 60% of PCOS women and 30% of non-PCOS women had FI. Around the world, FI is one of the most challenging conditions, especially in women, occurs as a consequence of nonsufficient or restricted availability of safe food resources, which can exert several adverse effects, such as obesity [30, 31, 54–56] and reduced physical activity [57]. Furthermore, obesity itself which can be manifested as high waist circumference can amplify the severity of metabolic disorders, such as PCOS [58–60]. Also, it is reported that physical activity is inversely associated with PCOS [8, 61]. So, it may be plausible that FI can indirectly affect PCOS risk. Our results demonstrated that PCOS risk was positively associated with waist circumference and menstrual age and negatively associated with physical activity. However, some other studies couldn’t find any significant association between PCOS with waist circumference, menstrual age [62], and physical activity [37, 62, 63] compared to non-PCOS women.

Several studies demonstrated a positive association between low economic levels with FI [64, 65] and PCOS [8, 32] in line with the present study. FI can induce persons to pay less cost for purchasing foods, consume smaller amounts of foods, change their dietary patterns [66–68], reduce the variety of dietary intakes, and increase the consumption of high-calorie foods [69]. A high-calorie diet itself can lead to hyperlipidemia, obesity, and T2DM [70–72].

According to the current study results, the quasi-western and healthy dietary patterns were associated with the increased and decreased risk of PCOS, respectively. This positive effect of quasi-western dietary patterns on the PCOS risk can be due to the low amount of healthy foods, including fruits and vegetables, and the high amount of unhealthy food items, such as meats, industrial juice, French fries, and sweets [40] and excessive rates of fats and sugar in this type of diet [73].

As an undeniable component of the quasi-Western diet, the saturated fatty acid is directly related to increased IR [74, 75] unlike omega-3 unsaturated fatty acid as a component of the healthy diet [2, 22]. This study identified higher consumption of saturated fats and MUFAs and lower consumption of cholesterols and docosahexaenoic acid in PCOS women. Numerous studies have shown that high dietary fat intakes, particularly trans- and saturated fats, are associated with higher risks of T2DM and CVD [76, 77], which can adversely affect the PCOS by increasing the inflammatory factors [78]. Our study found no significant differences between the intake of calories, trans fats, PUFAs, linoleic acid, linolenic acid, and eicosapentaenoic acid among women with and without PCOS. Douglas et al., in a cohort study on 30 PCOS women and 27 non-PCOS healthy women, indicated that total, trans- and saturated fats, MUFAs, PUFAs, and cholesterol were not significantly different between the groups [79]. However, other studies reported a higher intake of saturated fatty acids in PCOS women [80, 81]. Meats have been related to obesity and inflammation due to their high-fat content [82]. However, since iron deficiency is common in PCOS women [83], meats are suggested to be considered healthy dishes due to their high iron contents [61]. Our results demonstrated that adult women with PCOS were more adhere to meat consumption. In contrast, Hajivandi et al., in a qualitative study, reported a low intake of meats in overweight and obese adolescents with PCOS [61].

The association between high intake of protein with insulin and glucose responses is inconsistent [84, 85]. In the current study, the protein intake of PCOS women was lower; while the proportion of unhealthy protein, such as meats, to healthy protein sources, such as fish, poultry, legumes, and soy was higher in the PCOS women than in healthy women. However, other studies did not report any differences among women with and without PCOS [63, 79]. Based on a possible mechanism, animal proteins compared to vegetable proteins may increase the serum levels of insulin-like growth factor I (IGF-I) that can be involved in increasing the PCOS risk [8].

It was found that a high intake of fats and a low intake of carbohydrates can be positively associated with weight, insulin and androgen concentrations, and the prevalence of PCOS [86]. This study indicated that the total mean of carbohydrates was lower in women with PCOS than non-PCOS women. However, other studies showed no significant differences [63, 79]. On the other hand, our results demonstrated that the proportion intake of simple carbohydrates (sugar and soft drinks) to complex carbohydrates (legumes) was higher in the PCOS women. Our results reported that PCOS women were more likely to have high GI foods, and there was a positive association between this diet and IR [87]. Low GI foods appeared to have beneficial effects on IR improvement [88].

Several studies have demonstrated the inverse relationships between the healthy dietary intake, which is rich in fruits and vegetables with visceral fat, weight, and the risk of T2DM, and the intake of dairy products with IR and dyslipidemia [89–91]. This study showed that PCOS women received a lower intake of vegetables, fruits, and dairy products. As significant sources of fiber, vitamins, and minerals, Fruits and vegetables are associated with a lower prevalence of the metabolic disease [92]. It has been found that fiber intake is associated with a reduction of PCOS risk [81]. Moreover, dietary intake of dairy products exerts beneficial effects on the infertility and BMI by reducing the IR [93–95]. However, the effects of high-fat over low-fat dairies on PCOS are not well apparent [7]. Similar to the previous studies [37, 62], PCOS women in the present study had lower consumption of dairy products than non-PCOS women. However, Shishehgar et al., in a case-control study on 142 Iranian women with PCOS and 140 healthy women with normal menstrual patterns found no significant differences between the dietary intake of dairies and fruits among the groups [96].

The results of the current study demonstrated that PCOS women had a lower intake of several kinds of vitamins, such as A, B_5_, B_6_, B_12_, C, and D, potassium, iron, zinc, thiamine, niacin, magnesium, phosphorus, and total fiber compared to healthy women. It has been reported that adult women with FI less adhered to intake magnesium, vitamins B_6_ and E, thiamine, and niacin [97]. Consistent with our findings, Moran et al. in a population-based observational study, demonstrated a higher intake of magnesium, vitamins A and E, phosphorus, and iron in PCOS women [98]. Moreover, Douglas et al., found no significant differences in magnesium intake among women with and without PCOS [79]. Epidemiological studies have demonstrated that magnesium intake might decrease the risk of T2DM in PCOS women [99, 100], and dietary sodium intake can increase blood pressure [101]. Calcium is one of the crucial minerals due to its beneficial effects on IR, follicular maturation, and menstrual regulation, suggested to be consumed by PCOS women [24]. Several studies have indicated the role of vitamin D and calcium in IR and insulin secretion [102, 103]. However, the exact molecular mechanisms of the effects of vitamin D on the improvement of IR and PCOS are not yet clear [104]. The possible role of folate and vitamin B_12_ is decreasing the serum levels of homocysteine in PCOS women with IR [105, 106], and also zinc is modifying the clinical and biochemical factors of PCOS women [11]. In our study, there were no differences between the rates of trans fats, PUFAs, eicosapentaenoic acid, sodium, vitamin E, alpha-tocopherol, manganese, selenium, and chromium among PCOS and non-PCOS women. However, Eslamian et al., in a case-control study on 281 PCOS women and 472 healthy women aged 20-35 years, demonstrated the positive association between fat, animal protein, carbohydrate, cholesterol, saturated fatty acid, sodium, biotin, iron, copper, fluoride, zinc, and calcium with the PCOS risk [19].

Despite several studies, the optimal dietary components for PCOS are not well clear [107]. However, lifestyle management with dietary modifications is considered one of the first-line therapies for metabolic syndrome in overweight and obese women with PCOS. Aside from lifestyle management, treatment should be managed appropriately for each patient upon to their phenotype, signs, and symptoms [83].

Given the high importance of insulin resistance and compensatory hyperinsulinemia in the management of PCOS [108, 109], a study on the different forms of fasting, including intermittent fasting and periodic fasting, showed the significant decrease of IGF-1, IGFBP1, glucose and insulin levels, and consequently beneficial effects on ovarian function, androgen excess, and infertility in PCOS women [109].

In another study, Ramadan fasting in women with PCOS improved the plasma nitric oxide and glutathione levels without affecting glucose indices, lipids, and total antioxidant capacity [110].

So, further studies are requested to understand the exact mechanisms of how modifying lifestyle, especially dietary patterns, may be an important strategy for reducing PCOS.

Several significant strengths are in the present study. First, this study investigated the association between FI and PCOS for the first time in Iran. Second, we compared characteristics of both PCOS and non-PCOS women due to the case-control design of our study. Third, we considered the non-smoking women and excluded those who had any current history of cardiovascular, liver, and kidney diseases which might affect the results. Last but not least, this study also investigated the association between PCOS with dietary patterns and food intakes. However, our study had some limitations. First, the sample was limited to overweight and obese women with specific ranges of age and BMI, who were referred to the infertility clinic of one hospital, which makes it difficult to generalize the results to other women with other ages and BMI. Second, the different phenotypes of PCOS, the associations between metabolic and hormonal indices with FI and PCOS, the association between PCOS with fasting and dietary restrictions, and the altered metabolic pathways in PCOS were not assessed.

## Conclusion

In general, this case-control study has shown that the PCOS risk was positively associated with FI, quasi-western dietary patterns, waist circumference, menstrual age, and low economic levels and negatively associated with the healthy dietary patterns and physical activity even after controlling the potential confounders. Further prospective studies, including other ages and BMI, are required to confirm our findings and increase our understanding of the association between food security status and dietary patterns with PCOS.

## Data Availability

The datasets that were used and/or analyzed during the current study are available from the corresponding author on a reasonable request.

## Abbreviations

PCOS: Polycystic ovary syndrome
PCOM: Polycystic ovarian morphology
IR: Insulin resistance
T2DM: Type 2 diabetes
CVD: Cardiovascular disease
FI: Food insecurity
DASH: Dietary approaches to stop hypertension
BMI: Body mass index
MD: Mediterranean diet
GI: Glycemic index
kg/m^2^: Kilogram per meter square
cm: Centimeters
MET: Metabolic equivalents
USDA: United States Department of Agriculture
FFQ: Food frequency questionnaire
SD: Standard deviation
MUFAs: Monounsaturated fatty acids
PUFAs: Polyunsaturated fatty acids
ORs: Odds ratios
CIs: Confidence interval
IGF-I: Insulin-like growth factor I
